# Impact of Rehabilitation Nutrition and Healthy Weight Maintenance in Motor-Complete Tetraplegia Patients

**DOI:** 10.3390/jcm11174970

**Published:** 2022-08-24

**Authors:** Ji Cheol Shin, Kye Hee Cho, Eun Young Han, Kwang Ho Ahn, Sang Hee Im

**Affiliations:** 1 Department and Research Institute of Rehabilitation Medicine, Severance Hospital, Yonsei University College of Medicine, Seoul 03722, Korea; 2 Department of Rehabilitation Medicine, CHA Ilsan Medical Center, CHA University School of Medicine, Goyang 10414, Korea; 3 Department of Rehabilitation Medicine, Jeju National University School of Medicine, Jeju 63243, Korea

**Keywords:** spinal cord injury, motor-complete tetraplegia, malnutrition, low muscle mass, obesity

## Abstract

Cachexia and low muscle mass in motor-complete tetraplegia are associated with poor outcomes. This study aimed to document anthropometric, body composition, and nutritional indices in patients and to assess the effect of a comprehensive rehabilitation nutrition program in cachexia and low muscle mass. For 34 motor-complete tetraplegia in the subacute phase, a comprehensive rehabilitation nutrition program was provided for 8 to 9 weeks. Risk of malnutrition, anthropometric and body composition indices, as well as laboratory tests were assessed upon admission and at discharge. A body mass index of less than 20.2 kg/m^2^ was used as the cut-off value for obesity. Upon admission, 73.5% of patients were classified as obese, half were at risk of malnutrition, and 29.4% were compatible with cachexia. Compared to the premorbid state, the non-obese group showed greater weight reduction (*p* < 0.01) and higher prevalence of low muscle mass (*p* = 0.004) than the obese group. Disease duration was different between groups (*p* < 0.01). After rehabilitation, malnutrition risk, protein levels, and hemodynamic results improved in both groups (*p* < 0.05). A significant time × group interaction was observed for muscle mass, skeletal muscle mass, and appendicular lean mass index (*p* < 0.05). Muscle mass was maintained while fat components increased in both groups. Functional improvement was negatively correlated with an increase in fat components (*p* < 0.05). A personalized rehabilitation nutrition intervention improved the nutritional status, body composition, and functional outcomes in motor-complete tetraplegia. The increase in muscle mass was associated with functional gain; healthy weight gain or maintenance may improve the level of independence. Further studies to endorse this beneficial evidence of rehabilitation nutrition in the maintenance of muscle component are needed.

## 1. Introduction

Spinal cord injury (SCI) is a devastating condition that causes significant morbidity, disability, and dependency, limiting psychosocial behaviors. The American Spinal Injury Association Impairment Scale (AIS) defines the extent of SCI: Grade A denotes an absence of all motor and sensory functions distal to the site of injury; Grade B implies having some sensory function but no motor function; Grade C injuries have a motor grade of less than 3 below the neurological level; Grade D injuries have a motor grade of at least 3 below the neurological level [1].

Patients with motor-complete cervical SCI (AIS A and B) have the worst functional outcomes and achieve little neurological recovery [2]. This type of injury substantially decreases estimated life expectancy, with a cumulative 25-year survival rate of 38.1% for C1–C4 tetraplegia, 55.7% for C5–C8 tetraplegia ABC patients, and 70.4% for AIS/Frankel grade D patients with any level of injury [3]. Furthermore, the lifetime economic burden due to complete tetraplegia is more than twice that the one due to incomplete paraplegia [4].

Immediately after SCI, increased metabolic demand related to trauma and reduced caloric intake can aggravate weight loss [5]. SCI patients are then exposed to secondary causes of low muscle mass, including physical inactivity and malnutrition [6]. Disuse, spasticity, and microvascular damage contribute to atrophy and adipocyte accumulation below the level of injury [7]. Consequently, SCI patients are at high risk of neurogenic obesity and low muscle mass [5], predisposing them to cardiovascular diseases [8]. Patients with a higher level of cervical SCI exhibit a greater degree of cardiovascular dysfunction [9]. Moreover, a prospective cohort study of traumatic SCI patients reports that low muscle mass predicts early post-injury mortality and acute care adverse events [10].

Rehabilitation nutrition, defined as nutrition care management occurring within the context of rehabilitation [11], has recently been introduced. Attempts to determine the associations between nutritional support and rehabilitation outcomes have been established for sarcopenia [12], stroke [13], cancer, and hip fracture [14]. However, SCI is a more complex disability with a multitude of factors contributing to an inadequate nutritional status. Although some studies have assessed exercise-based interventions to address low muscle mass and obesity, no study has yet reported the effect of nutritional intervention on the management of these conditions in SCI patients.

This study aimed to characterize anthropometric and body composition indices measured via bioelectrical impedance analysis, malnutrition risk screening, and laboratory studies of obese and non-obese motor-complete tetraplegia in the subacute stage. Additionally, the effect of a comprehensive rehabilitation nutrition program implemented during conventional rehabilitation therapy on these indices and its functional outcomes were investigated.

## 2. Materials and Methods

### 2.1. Study Population

This study is a retrospective review of motor-complete tetraplegia patients. Among 254 SCI patients admitted to a tertiary university hospital for short-term (8 to 9 weeks) rehabilitation treatment between January 2017 and August 2018, motor-complete tetraplegia (AIS grade A and B, *n* = 34) patients were selected. Their medical records, laboratory tests, and nutritional consultations were reviewed and only those with available results of below neurological level assessments were included. A post-injury duration of 2 to 8 weeks was classified as subacute [15] and ≥8 weeks was classified as late-subacute.

### 2.2. Measurement of Body Mass Index and Body Composition Analysis

Patients were weighted using a digital wheelchair scale (Kyongin Medical, Seoul, Korea), and total body weight was calculated by subtracting the weight of the wheelchair. Body weight prior to injury was documented at admission based on the patient’s recollection and was included as data in addition to body weight upon admission and at discharge. Patients with a ≥5% reduction in body weight over the previous 12 months compared with that prior to SCI, as well as three of the following criteria, were classified as having cachexia. The criteria were (1) decreased muscle strength, (2) fatigue, (3) anorexia, (4) low fat-free mass index, and (5) abnormal biochemistry: C-reactive protein (CRP) (>5 mg/L), IL-6 (>4 pg/mL), anemia (hemoglobin < 12 g/dL), low serum albumin (<3.2 g/dL) [16]. The body mass index (BMI) was calculated as weight (kg) divided by known height (m^2^). As BMI cutoff values for obesity depend on sex, race, and the specific population, a lower cutoff value of 20.2 kg/m^2^ for motor-complete SCI patients in Korea [17] was used rather than the 22.0 kg/m^2^ of universal SCI [18].

Body composition was measured using a multifrequency bioelectrical impedance analyzer (Inbody S10, Seoul, Korea) [19] by trained staff according to standardized procedures. After emptying the bladder, the patients remained supine for 10 min prior to measurement for even body water dispersion. The arms were placed 15 degrees away from the trunk, and the legs were placed shoulder width apart so that the thighs did not make contact. Electrodes were attached to both the hands and the legs. Indices of bioelectrical impedance analysis (BIA) included total muscle mass (kg), skeletal muscle mass (kg), appendicular lean mass (kg), fat mass (kg), and fat percentage (%). Appendicular lean mass and fat mass divided by height (m^2^) was defined as the appendicular lean mass index (ALMI) and fat mass index (FMI), respectively. The cutoff value for low muscle mass based on BIA was defined as ALMI < 7.0 kg/m^2^ for men and <5.7 kg/m^2^ for women [20].

### 2.3. Screening of Nutritional Status and Nutritional Program

To assess nutritional status, laboratory tests (i.e., complete blood count with differentials, electrolytes, liver and kidney function test, serum protein, albumin, CRP, as well as lipid panel and nutritional data) were conducted. The nutritional risk screening test, utilizing serum albumin level, age, hematocrit (%), and total lymphocyte count as reference values, was used to screen the malnutrition risk within 24 h of admission, with a score of >3.5 indicating a malnutrition risk [21].

Personalized nutrition intervention was provided by a professional nutritionist. Nutrition assessment included anthropometric measurements, laboratory tests, medical history, dietary pattern, and level of physical activity. The daily energy requirement (kcal) was calculated by body weight (kg) × energy depending on the activity level (kcal/kg). For this calorie calculation, actual body weight was used for non-obese patients while adjusted body weight was used for obese patients. Ideal body weight (IBW), which is adequate for the maintenance of health, was estimated as height squared × 22 [22]. The adjusted body weight was calculated using formula below.

(1) $$\mathrm{Ajusted}\;\mathrm{body}\;\mathrm{weight}=\mathrm{IBW}+0.25\left(\mathrm{actual}\;\mathrm{body}\;\mathrm{weight}-\mathrm{IBW}\right)$$

During admission, weekly body weight assessment and monthly body composition analysis were performed to monitor the adequacy of the dietary plan. The long-term goal of the intervention was the progressive achievement of IBW within 6 months to one year.

Since motor-complete tetraplegia patients lack physical activity, 20 to 23 kcal/kg/day was recommended with 15% to 20% of calorie reduction [23,24]. For patients with a high risk of malnutrition, protein and energy-enriched/fortified meals and supplementation were provided for depleted nutrition. By monitoring oral intake, additional parenteral nutrition was provided for those consuming less than 75% of the nutritional requirements.

### 2.4. Functional Assessments

Functional assessment was conducted using the Modified Barthel Index (MBI), the Functional Independence Measure (FIM), and the Spinal Cord Independence Measure (SCIM). MBI is a 100-point rating scale for the completion of nine self-care and six mobility tasks, each assigned a numeric value according to levels of assistance [25]. FIM is an 18-item, 7-level scale assessing the severity of the disability and the medical rehabilitation functional outcome [26]. SCIM assesses functioning in activities of daily living including burden of care, and achievements of medical, psychological, or social relevance in SCI, in a 100-point score grouped into three subscales of self-care, respiration, and sphincter management, as well as mobility [27]. SCIM has been revised into a second version (SCIM 2) to raise sensitivity to functional changes [28], and then to a third version (SCIM 3) to overcome intercultural differences [29].

### 2.5. Conventional Rehabilitation Therapy

Based on a comprehensive evaluation of function, body composition, and nutritional status, a comprehensive rehabilitation program was provided for 8 to 9 weeks of in-hospital rehabilitation.

Daily physical therapy was conducted by experienced physical therapists for SCI and included the reconditioning of non-paralyzed body parts and the correction of paralysis-induced agonist–antagonist co-contraction imbalance via neuromuscular re-education. Depending on the individual functional level, the training tasks consisted of rolling, sitting balance training, transfer, passive, and active range of motion exercise, active or assistive strengthening exercises, and assisted standing on a tilt table while monitoring autonomic nervous system symptoms, such as faintness and pain. Physical therapy was conducted for ≥30 min sessions twice a day for more than 5 days a week. Functional electrical stimulation (FES) was conducted for at least 20 min daily until the intended joint movement was achieved or visible muscle contraction was induced. The portable FES unit (Walking Man II; Cyber Medic, Iksan, Korea) was set at 24 Hz with a pulse width of 150 µs, asymmetrical biphasic waveform, and maximal intensity of 90 mA. Upon admission, a physiatrist and a physical therapist educated the patient and caregiver on respiratory muscle training so that the exercises could be repeated 2 to 3 times daily by themselves at the bedside.

Occupational therapy consisted of assistive upper limb stretching exercises to prevent contractures and maintain muscle strength and endurance as well as task-oriented therapy for activities of daily living. The proper use of an environmental control unit, orthotics, and splints for assisting hand function, transfer, and wheelchair manipulation were also taught after wheelchair adjustments were made.

### 2.6. Statistical Analysis

Data were analyzed using standard statistical software (SPSS version 21.1 for Windows, SPSS, Inc., Chicago, IL, USA). Categorical variables were analyzed by χ^2^ tests. Differences in anthropometric and body composition values between obese and non-obese groups as well as before and after rehabilitation nutrition were analyzed using Mann–Whitney U tests and Wilcoxon signed-rank tests, respectively. Repeated measures ANOVA was used to test the main effect of time and time × group interaction. A *p*-value < 0.05 was considered statistically significant. The statistical power of the data was confirmed by G* Power analysis [30]. Pearson correlation coefficients were calculated to determine the relationship between changes in body composition and functional measures. Values are provided in mean value ± standard deviation or number with percentage.

## 3. Results

**Figure 1:**
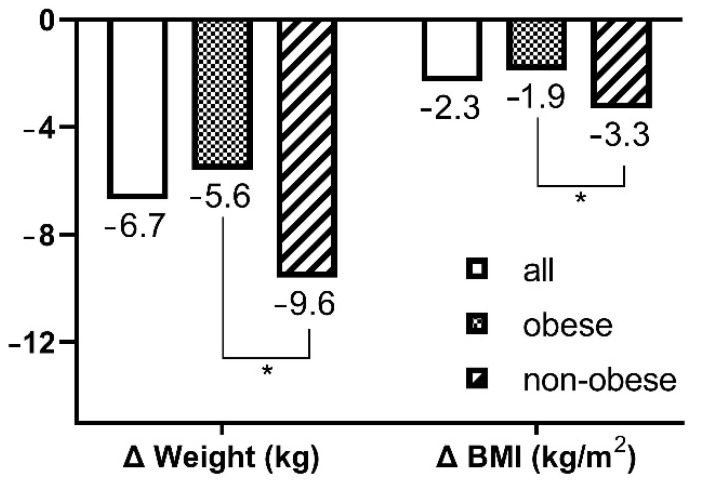
Change of body weight and body mass index between premorbid state and at admission. * *p* < 0.05 between groups comparison by Mann–Whitney test. Δ, change; BMI, Body Mass Index.

Of the 34 motor-complete SCI patients, most were men (*n* = 33, 97.1%) and AIS grade A (*n* = 23, 67.6%). The average disease duration was 71.0 days, with the non-obese group (124.8 ± 86.6) having a significantly longer duration than the obese group (55.6 ± 38.6; *p* < 0.01). The weight and BMI of all patients at admission were significantly lower than that of the premorbid state (*p* < 0.01); however, 73.5% of patients (*n* = 25) were classified as obese. Weight and BMI were higher in the obese group than in the non-obese group before the injury and upon admission (*p* < 0.05; Table 1). However, the reduction in weight and BMI since the injury was significantly greater in the non-obese group (−9.6 ± 5.4 kg and −3.3 ± 1.8 kg/m^2^) than in the obese group (−5.6 ± 4.4 kg and −1.9 ± 1.5 kg/m^2^, *p* < 0.05: Figure 1).

At admission, half of the patients (*n* = 17) were at risk of malnutrition, and 29.4% of the patients (*n* = 10) were compatible with cachexia. The prevalence of cachexia was significantly different (*p* = 0.004) between groups; four (16.0%) in the obese and six (66.7%) in the non-obese group. Upon admission, six (24.0%) of the obese group and seven (77.8%) of the non-obese group had low muscle mass (*p* = 0.004, Table 2). At discharge, three of seven patients in the non-obese group recovered from ‘low muscle mass’ status, whereas three obese patients newly developed low muscle mass. The overall prevalence of obesity increased from 73.5% to 79.4% between admission and discharge, and 44.4% of the non-obese (four of nine) patients became obese. Using low ALMI < 7.0 kg/m^2^ as the reference to sarcopenia, the prevalence of sarcopenic obesity tended to increase in both groups (Table 2); initially, sarcopenic obese patients accounted for 17.6% (*n* = 6), which then increased to 35.3% (*n* = 14) at discharge in all patients.

Half of the patients were at high risk of malnutrition and the mean levels of basal CRP, protein, and hematocrit were abnormal. However, the personalized nutrition intervention reduced the prevalence of malnutrition in both groups, and the levels of albumin, protein, hemoglobin, and hematocrit were improved in all patients, especially in the obese group (*p* < 0.05; Table 2).

At discharge, weight and BMI did not change significantly. However, fat mass, fat percentage, and FMI increased regardless of obesity (*p* < 0.01, Table 3). An increase in muscle mass, skeletal mass, and ALMI was observed in the non-obese group, and repeated measures ANOVA revealed significant changes in muscle components after rehabilitation depending on the obesity group (*p* < 0.05). Modest improvements in functional assessments (i.e., MBI, FIM, SCIM2, and SCIM3) were noted after rehabilitation (Table 3).

Changes in weight and BMI were positively correlated with functional improvement based on FIM (*p* < 0.05, Table 4). By contrast, changes in fat mass, fat percentage, and FMI were negatively correlated with functional improvement based on SCIM2 (*p* < 0.05, Table 4).

## 4. Discussion

We found that patients with motor-complete tetraplegia experienced severe weight loss and malnutrition, cachexia, low muscle mass, and obesity during the subacute and late-subacute phases. However, a comprehensive rehabilitation nutritional intervention reversed this process.

SCI patients undergo a catabolic process that results in protein store depletion and altered fat metabolism that leads to malnutrition and imbalance of fat and muscle components [31]. These changes were made evident in our study through serial laboratory tests and body composition assessments. Patients before the injury had a body weight and BMI similar to that of age-matched healthy men [32]. However, after the injury, the weight and BMI were reduced (24.2 ± 2.7 vs. 22.0 ± 2.7 kg/m^2^ and 71.9 ± 9.4 vs. 65.2 ± 9.1 kg) while fat mass and fat percentage tended to increase, which may suggest that the weight reduction was mostly due to the loss of muscle mass. Although premorbid weight, BMI, and most of body composition parameters were lower in the non-obese group than in the obese group, the reduced amount between the injury and the admission was greater in the non-obese group. Interestingly, there was a significant time × group interaction on muscle mass, skeletal mass, and ALMI between groups (*p* < 0.05) with the non-obese group exhibiting higher values after short-term rehabilitation nutrition. This finding was also comparable with the reduction in the proportion of low muscle mass in the non-obese group. It appears that the rehabilitation nutrition may warrant a chance to minimize muscle loss, especially in the non-obese patients.

In complete SCI patients, a further atrophy of up to 27 to 56% takes place between 6 and 24 weeks post-injury [33]. In this study, the obese group had a shorter disease duration (55.6 days) than the non-obese group (124.8 days; *p* < 0.01). Thus, SCI-induced muscle atrophy may have been ongoing in the obese group. A loss of muscle mass was observed more in the obese group, whereas muscle mass was relatively preserved in the non-obese group. The clinical implication of this study is to suggest the means to gain or maintain muscle mass in complete tetraplegia patients who can hardly do so independently. As subacute patients showed different tendencies, the effect of the rehabilitation nutrition may vary depending on the presence of obesity and disease duration.

Regardless of obesity, there were no significant changes in body weight and BMI after short-term rehabilitation nutrition. By contrast, a previous review of weight change during the first-year post-SCI reported a BMI decrease among overweight and obese patients, and an increase among normal or underweight patients [5]. This study consisted of heterogeneous groups of SCI severity and levels, used a higher BMI cutoff for obesity (>25 kg/m^2^), performed a follow-up at one year after the injury instead of immediately after rehabilitation, and did not conduct body composition and/or functional assessments [5].

The group differences in muscle components could have been related to FES use. It is a confirmed method for muscle mass maintenance in SCI or paralysis-bound patients who cannot actively contract muscles or who are at high risk of muscle atrophy. In this study, the non-obese group with less fat tissue interference [34] may have benefited more from FES for the contraction of paralyzed muscles than the obese group. In contrast to the muscle-related parameters, fat components including fat mass, fat percentage, and FMI increased during intervention regardless of obesity. A systematic review of obesity management in SCI patients concludes that neuromuscular electrical stimulation increases lean body mass but does not affect weight or total fat mass [35]. However, a recent study of FES cycling and nutritional counseling reports a significant decrease in body fat with muscle gain in obese SCI patients [36]. Further studies of nutritional and anabolic rehabilitation approaches for SCI with baseline fat content may clarify the effect of FES on BIA indices.

Due to the catabolic process causing protein store depletion and alterations in fat metabolism, SCI patients are at risk of malnutrition [31]. Additionally, a diminished resting metabolic rate results in excess energy intake and subsequent obesity as well as obesity-related co-morbidities [23]. It is also reported that individuals with SCI have an average of 13% more fat than non-SCI controls for any given BMI [37] and that malnutrition and being overweight are highly prevalent in SCI [31].

The rehabilitation nutrition program contributed to significant improvements in abnormal laboratory findings at discharge. Levels of protein, hemoglobin, and hematocrit, which are predictors of malnutrition and disease prognosis [21], improved. Likewise, the proportions of patients at risk of malnutrition decreased in both groups. Increased CRP and low levels of total lymphocyte count may reflect inflammatory [38] and secondary immunosuppression [39] in early stages and high-level SCI, respectively. The level of serum albumin also declines in the presence of inflammation [40]. At discharge, the levels of CRP decreased, while the total lymphocyte count and the level of albumin increased, although the changes were not significant. The personalized nutritional intervention might be effective in reversing the course of malnutrition and bring positive body composition changes.

After rehabilitation nutrition, modest improvements in functional assessments were noted, although the actual degree of functional gain is difficult to determine. Improved SCIM scores were negatively correlated with an increase in fat components, whereas improved FIM scores were positively correlated with amount of weight and BMI gain. This “healthy” weight gain is likely to be the result of an increase in fat-free mass. Similar to a retrospective cohort of cervical SCI in which a good nutritional state was associated with a high functional independence [41], the slight yet significantly improved SCIM may suggest a functional benefit of the rehabilitation nutrition and healthy weight gain.

This study has several limitations to consider in the interpretation of the results. First, contributing factors such as total amount of food intake and/or extra calorie intake could not be assessed due to the retrospective nature of the study. However, total nutritional intake was likely to be well-controlled and consistent with the dietary plan as patients were hospitalized. Second, the cause-and-effect relationships between the parameters associated with disease prognosis or mortality were not investigated. Third, the patients’ selection may be biased due to a single-center study with most of the patients being male. However, many SCI studies mainly consist of male participants due to the gender characteristics of this condition. Fourth, the small number of patients, especially in the non-obese group, could be a limitation, although it is difficult to recruit motor-complete SCI patients. Lastly, the method for malnutrition assessment still included albumin which has been recommended not to be used as a diagnostic marker. Further studies should be executed with updated assessments for malnutrition excluding albumin.

## 5. Conclusions

In conclusion, this study confirms the high prevalence of low muscle mass, obesity, malnutrition, and cachexia in patients with motor-complete tetraplegia. To the best of our knowledge, this is the first study to demonstrate the beneficial effect of a nutritional intervention incorporated into rehabilitation therapy on nutritional and functional outcomes, as well as muscle mass preservation. Various body composition indices differed depending on obesity; healthy weight gain or maintenance was associated with functional improvement. Rehabilitation nutrition needs to be further investigated in relation to healthy weight gain and fat reduction in large-scaled prospective studies to determine their long-term effects on disease prognosis and mortality.

## Figures and Tables

**Table 1:** General and clinical characteristics of the obese and non-obese groups.

	All (*n* = 34)	Obese (*n* = 25)	Non-Obese (*n* = 9)	Intergroup *p* Value
Age (year, range)	45.7 ± 16.9	46.8 ± 17.2	42.6 ± 16.7	0.530
Male/female	33/1	24/1	9/0	0.543
AIS grade A/B	23/11	18(72.0)/7(28.0)	5(55.6)/4(44.4)	0.366
Disease duration (days)	47.5 (16–244)	38 (16–157)	79 (19–244)	0.002 *
Rehab duration (days)	52.5 (20–172)	54 (20–172)	50 (30–125)	0.483
Subacute/late-subacute (%)	18/16	16 (64.0)/9 (36.0)	2 (22.2)/7 (77.8)	0.031 *
Height (cm)	172.1 ± 5.5	172.1 ± 5.5	172.0 ± 5.8	0.956
Weight (kg)				
Premorbid	71.9 ± 9.4	74.4 ± 8.7	64.9 ± 7.8	0.007 **
At admission	65.2 ± 9.1 ^†††^	68.8 ± 7.1 ^†††^	55.2 ± 6.0 ^††^	<0.001 ***
BMI (kg/m^2^)				
Premorbid	24.2 ± 2.7	25.1 ± 2.3	21.9 ± 2.6	0.001 **
At admission	22.0 ± 2.7 ^†††^	23.2 ± 1.8 ^†††^	18.6 ± 1.9 ^††^	<0.001 ***
Body composition				
Fat (kg)	16.1 ± 5.1	17.4 ± 5.0	12.4 ± 3.5	0.008 **
Fat (%)	24.5 ± 6.5	25.4 ± 6.7	22.1 ± 5.3	0.208
FMI (kg/m^2^)	5.5 ± 1.8	5.9 ± 1.8	4.2 ± 1.2	0.010 *
Muscle mass (kg)	46.4 ± 7.0	48.5 ± 6.5	40.4 ± 4.3	0.002 **
Skeletal muscle mass (kg)	26.2 ± 4.3	27.5 ± 4.1	22.6 ± 3.0	0.002 **
Skeletal muscle mass (%)	40.4 ± 3.6	40.3 ± 3.7	40.6 ± 3.5	0.837
ALMI (kg/m^2^)	7.5 ± 1.3	7.9 ± 1.1	6.2 ± 0.7	<0.001 ***
Functional measures				
MBI	2.2 ± 5.1	1.9 ± 3.8	3.3 ± 8.4	0.821
FIM	49.4 ± 3.3	49.2 ± 2.8	50.0 ± 4.9	0.984
SCIM2	12.6 ± 4.6	12.8 ± 3.8	12.4 ± 6.9	0.757
SCIM3	12.4 ± 4.3	12.6 ± 3.6	12.1 ± 6.3	0.757
Comorbidities (*n*)				
Diabetes mellitus	2	1	1	
Hypertension	5	5	0	
Dyslipidemia	2	1	1	

Values are mean ± standard deviation or numbers (%), except for duration which is median (range). * *p* < 0.05, ** *p* < 0.01, *** *p* < 0.001 for intergroup comparison by ANOVA, ^††^ *p* < 0.01, ^†††^ *p* < 0.001 by Wilcoxon signed-rank test. AIS, American Spinal Injury Association impairment scale; ALMI, Appendicular Lean Mass Index; BMI, Body Mass Index; FMI, Fat Mass Index; MBI, Modified Barthel Index; FIM, Functional Independence Measure; SCIM, Spinal Cord Independence Measure.

**Table 2:** Change of nutritional status in the obese and non-obese groups.

	All (*n* = 34)	Obese (*n* = 25)	Non-Obese (*n* = 9)	Intergroup *p* Value
Cachexia	10 (29.4)	4 (16.0)	6 (66.7)	0.004 *
Low muscle mass				
initial	13 (38.2)	6 (24.0)	7 (77.8)	0.004 *
f/u	13 (38.2)	9 (36.0)	4 (44.4)	0.248
Obesity				
initial	25 (73.5)	25 (100.0)	0 (0.0)	-
f/u	27 (79.4)	23 (92.0)	4 (44.4)	0.002 **
Sarcopenic obesity				
initial	6 (17.6)	6 (24.0)	0 (0.0)	0.105
f/u	13 (35.3)	11 (44.0)	2 (22.2)	0.249
Risk of malnutrition (NRST > 3.5)				
initial	17 (50.0)	11 (44.0)	6 (66.7)	0.244
f/u	5 (14.7) ^††^	3 (12.0) ^†^	2 (22.2) ^†^	0.458
Laboratory findings				
CRP (mg/dL)				
initial	23.1 ± 42.9	23.1 ± 48.8	22.8 ± 20.8	0.985
f/u	11.8 ± 15.3	9.6 ± 9.6	18.1 ± 25.1	0.157
Albumin (g/dL)				
initial	3.5 ± 0.4	3.5 ± 0.3	3.6 ± 0.4	0.311
f/u	3.7 ± 0.3 ^††^	3.7 ± 0.4 ^††^	3.7 ± 0.2	0.875
Protein (g/dL)				
initial	6.1 ± 0.5	6.0 ± 0.5	6.2 ± 0.6	0.425
f/u	6.3 ± 0.4 ^††^	6.3 ± 0.4 ^††^	6.2 ± 0.4	0.538
Hemoglobin (g/dL)				
initial	12.0 ± 1.3	12.0 ± 1.3	12.3 ± 1.4	0.518
f/u	12.8 ± 1.1 ^†††^	12.8 ± 1.1 ^††^	13.0 ± 1.2 ^††^	0.563
Hematocrit (%)				
initial	35.6 ± 3.5	35.4 ± 3.5	36.3 ± 3.8	0.491
f/u	37.9 ± 3.3 ^†††^	37.6 ± 3.2 ^††^	38.8 ± 3.8 ^†^	0.371
Total lymphocytes (cells/uL)				
initial	1657.9 ± 481.0	1664.4 ± 517.2	1640.0 ± 389.0	0.899
f/u	1766.5 ± 373.7	1793.6.5 ± 386.4	1691.1 ± 345.8	0.489

Values are mean ± standard deviation or numbers (%). * *p* < 0.05, ** *p* < 0.01 for intergroup comparison by χ^2^ tests. ^†^ *p* < 0.05, ^††^ *p* < 0.01, ^†††^ *p* < 0.001 for intragroup comparison by Wilcoxon signed-rank test. CRP, C-reactive protein; f/u, follow-up; NRST, Nutritional Risk Screening Test.

**Table 3:** Changes of anthropometric, body composition indices and functional outcome in the obese and non-obese groups.

	Initial	Follow Up	Δ	Time Effect	Time × Group Effect
				*p* Value	*p* Value
Weight (kg)				0.727	0.249
obese	68.8 ± 7.1	68.1 ± 8.4	−1.8 ± 8.8		
non-obese	55.2 ± 6.0 ^†††^	56.6 ± 4.8 ^†††^	1.0 ± 2.8		
BMI (kg/m^2^)				0.760	0.238
obese	23.2 ± 1.8	22.9 ± 2.1	−0.8 ± 1.7		
non-obese	18.6 ± 1.9 ^†††^	19.1 ± 1.7 ^†††^	1.1 ± 0.9		
Fat (kg)				0.006 **	0.437
obese	17.4 ± 5.0	19.3 ± 7.5	2.5 ± 4.5		
non-obese	12.4 ± 3.5 ^††^	15.5 ± 5.6	3.7 ± 3.6		
Fat (%)				0.003 **	0.537
obese	25.4 ± 6.7	28.8 ± 12.0	3.5 ± 7.4		
non-obese	22.2 ± 5.3	27.3 ± 9.4 ^†^	5.1 ± 4.4		
FMI (kg/m^2^)				0.005 **	0.482
obese	5.9 ± 1.8	6.6 ± 2.8	0.7 ± 1.7		
non-obese	4.2 ± 1.2 ^†^	5.3 ± 2.0	1.1 ± 0.9		
Muscle mass (kg)				0.455	0.022 *
obese	48.5 ± 6.5	45.6 ± 6.9	−1.7 ± 5.5		
non-obese	40.4 ± 4.3 ^††^	46.0 ± 8.5	3.4 ± 6.3		
Skeletal muscle mass (kg)				0.449	0.027 *
obese	27.5 ± 4.0	25.8 ± 3.9	−2.4 ± 4.4		
non-obese	22.6 ± 2.8 ^††^	26.0 ± 5.4	3.7 ± 3.6		
ALMI (kg/m^2^)				0.383	0.023 *
obese	7.9 ± 1.1	7.4 ± 1.5	−0.7 ± 1.8		
non-obese	6.2 ± 0.7 ^†††^	7.5 ± 1.9	1.1 ± 2.2		
Functional outcomes					
MBI				0.014 *	0.546
obese	1.9 ± 3.8	4.3 ± 5.7	2.3 ± 3.9		
non-obese	3.3 ± 8.4	4.8 ± 8.1	1.3 ± 2.2		
FIM				0.028 *	0.530
obese	49.2 ± 2.8	51.6 ± 6.3	0.4 ± 11.0		
non-obese	50.0 ± 4.9	51.4 ± 5.1	1.2 ± 2.0		
SCIM2				<0.001 **	0.812
obese	12.8 ± 3.8	16.0 ± 5.1	2.7 ± 4.7		
non-obese	12.4 ± 6.9	16.0 ± 7.3	3.2 ± 3.2		
SCIM3				<0.001 **	0.421
obese	12.6 ± 3.6	15.5 ± 4.6	2.4 ± 4.4		
non-obese	12.1 ± 6.3	16.3 ± 7.2	3.7 ± 3.6		

Values are mean ± standard deviation (SD) * *p* < 0.05, ** *p* < 0.01, comparison between initial and follow-up by repeated measure ANOVA, ^†^ *p* < 0.05, ^††^ *p* < 0.01, ^†††^ *p* < 0.001, comparison between groups by Kruskal–Wallis. ALMI, Appendicular Lean Mass Index; BMI, Body Mass Index; FIM, Functional Independence Measure; FMI, Fat Mass Index; MBI, Modified Barthel Index; SCIM, Spinal Cord Independence Measure; Δ, change.

**Table 4:** Correlation between change of body composition and functional measures.

	Δ SCIM2	Δ SCIM3	Δ FIM	Δ MBI
Δ Weight (kg)	0.009	0.005	0.394 *	0.201
Δ BMI (kg/m^2^)	0.012	0.006	0.397 *	0.209
Δ Fat (kg)	−0.342 *	−0.327	−0.074	0.104
Δ Fat (%)	−0.375 *	−0.359 *	−0.276	0.025
Δ FMI (kg/m^2^)	−0.343 *	−0.328	−0.069	0.116
Δ Muscle mass (kg)	−0.256	−0.224	−0.153	−0.018
Δ Skeletal mass (kg)	−0.230	−0.182	−0.167	−0.087
Δ ALMI (kg/m^2^)	−0.141	−0.082	−0.074	−0.050

Values are Pearson correlation coefficient. * *p* < 0.05, Pearson correlation analysis. Δ, change; SCIM, Spinal Cord Independence Measure; FIM, Functional Independence Measure; MBI, Modified Barthel Index; FMI, Fat Mass Index; ALMI, Appendicular Lean Mass Index; BMI, Body Mass Index.

## Data Availability

The data presented in this study are available upon request from the corresponding author. The data are not publicly available due to ethical issues.

## References

[1] van Middendorp J.J., Goss B., Urquhart S., Atresh S., Williams R.P., Schuetz M. (2011). Diagnosis and prognosis of traumatic spinal cord injury. Glob. Spine J..

[2] Khorasanizadeh M., Yousefifard M., Eskian M., Lu Y., Chalangari M., Harrop J.S., Jazayeri S.B., Seyedpour S., Khodaei B., Hosseini M. (2019). Neurological recovery following traumatic spinal cord injury: A systematic review and meta-analysis. J. Neurosurg. Spine.

[3] Savic G., DeVivo M.J., Frankel H.L., Jamous M.A., Soni B.M., Charlifue S. (2017). Long-term survival after traumatic spinal cord injury: A 70-year British study. Spinal Cord.

[4] Krueger H., Noonan V.K., Trenaman L.M., Joshi P., Rivers C.S. (2013). The economic burden of traumatic spinal cord injury in Canada. Chronic Dis. Inj. Can..

[5] Powell D., Affuso O., Chen Y. (2017). Weight change after spinal cord injury. J. Spinal Cord Med..

[6] Kim T.N., Choi K.M. (2013). Sarcopenia: Definition, epidemiology, and pathophysiology. J. Bone Metab..

[7] Scelsi R. (2001). Skeletal Muscle Pathology after Spinal Cord Injury: Our 20 Year Experience and Results on Skeletal Muscle Changes in Paraple-gics, Related to Functional Rehabilitation. Basic Appl. Myol..

[8] Garshick E., Kelley A., Cohen S.A., Garrison A., Tun C.G., Gagnon D., Brown R. (2005). A prospective assessment of mortality in chronic spinal cord injury. Spinal Cord.

[9] West C.R., Mills P., Krassioukov A.V. (2012). Influence of the neurological level of spinal cord injury on cardiovascular outcomes in humans: A meta-analysis. Spinal Cord.

[10] Banaszek D., Inglis T., Ailon T., Morin R.C., Dea N., Fisher C.G., Brain K.K., Scott J.P., John S. (2019). P153. Sarcopenia, independent of age, predicts mortality and acute care adverse events in patients with traumatic spinal cord injury. Spine J..

[11] Wakabayashi H., Sakuma K. (2014). Rehabilitation nutrition for sarcopenia with disability: A combination of both rehabilitation and nutrition care management. J. Cachexia Sarcopenia Muscle.

[12] Nagano A., Nishioka S., Wakabayashi H. (2019). Rehabilitation Nutrition for Iatrogenic Sarcopenia and Sarcopenic Dysphagia. J. Nutr. Health Aging.

[13] Shimazu S., Yoshimura Y., Kudo M., Nagano F., Bise T., Shiraishi A., Sunahara T. (2021). Frequent and personalized nutritional support leads to improved nutritional status, activities of daily living, and dysphagia after stroke. Nutrition.

[14] Nishioka S., Aragane H., Suzuki N., Yoshimura Y., Fujiwara D., Mori T., Kanehisa Y., Iida Y., Higashi K., Yoshimura-Yokoi Y. (2021). Clinical practice guidelines for rehabilitation nutrition in cerebrovascular disease, hip fracture, cancer, and acute illness: 2020 update. Clin. Nutr. ESPEN.

[15] Chen Q., Zheng W., Chen X., Li X., Wang L., Qin W., Li K., Chen N. (2019). Reorganization of the somatosensory pathway after subacute incomplete cervical cord injury. NeuroImage Clin..

[16] Evans W.J., Morley J.E., Argilés J., Bales C., Baracos V., Guttridge D., Jatoi A., Kalantar-Zadeh K., Lochs H., Mantovani G. (2008). Cachexia: A new definition. Clin. Nutr..

[17] Yun J.H., Chun S.M., Kim J.C., Shin H.I. (2019). Obesity cutoff values in Korean men with motor complete spinal cord injury: Body mass index and waist circumference. Spinal Cord.

[18] Laughton G.E., Buchholz A.C., Ginis K.A.M., Goy R.E. (2009). Lowering body mass index cutoffs better identifies obese persons with spinal cord injury. Spinal Cord.

[19] Panisset M.G., Desneves K., Ward L.C., Rafferty J., Rodi H., Roff G., El-Ansary D., Galea M.P. (2018). Bedside quantification of fat-free mass in acute spinal cord injury using bioelectrical impedance analysis: A psychometric study. Spinal Cord.

[20] Chen L.-K., Woo J., Assantachai P., Auyeung T.-W., Chou M.-Y., Iijima K., Jang H.C., Kang L., Kim M., Kim S. (2020). Asian Working Group for Sarcopenia: 2019 Consensus Update on Sarcopenia Diagnosis and Treatment. J. Am. Med. Dir. Assoc..

[21] Han J.S., Lee S.M., Chung H.K., Ahn H.S., Lee S.M. (2009). Development and Evaluation of a Nutritional Risk Screening Tool (NRST) for Hospitalized Patients. Korean J. Nutr..

[22] Tokunaga K., Matsuzawa Y., Kotani K., Keno Y., Kobatake T., Fujioka S., Tarui S. (1991). Ideal body weight estimated from the body mass index with the lowest morbidity. Int. J. Obes..

[23] Farkas G.J., Pitot M.A., Berg A.S., Gater D.R. (2019). Nutritional status in chronic spinal cord injury: A systematic review and meta-analysis. Spinal Cord.

[24] Academy of Nutrition and Dietetics (2009). Spinal Cord Injury (SCI) Evidence-Based Nutrition Practice Guideline.

[25] Granger C.V., Albrecht G.L., Hamilton B.B. (1979). Outcome of comprehensive medical rehabilitation: Measurement by PULSES profile and the Barthel Index. Arch. Phys. Med. Rehabil..

[26] Hamilton B.B., Laughlin J.A., Fiedler R.C., Granger C.V. (1994). Interrater reliability of the 7-level functional independence measure (FIM). Scand. J. Rehabil. Med..

[27] Anderson K., Aito S., Atkins M., Sorensen F.B., Charlifue S., Curt A., Ditunno J., Glass C., Marino R., Marshall R. (2008). Functional recovery measures for spinal cord injury: An evidence-based review for clinical practice and research. J. Spinal Cord Med..

[28] Itzkovich M., Tripolski M., Zeilig G., Ring H., Rosentul N., Ronen J., Spasser R., Gepstein R., Catz A. (2002). Rasch analysis of the Catz-Itzkovich spinal cord independence measure. Spinal Cord.

[29] Itzkovich M., Gelernter I., Biering-Sorensen F., Weeks C., Laramee M.T., Craven B.C., Tonack M., Hitzig S.L., Glaser E., Zeilig G. (2007). The Spinal Cord Independence Measure (SCIM) version III: Reliability and validity in a multi-center international study. Disabil. Rehabil..

[30] Faul F., Erdfelder E., Lang A.G., Buchner A. (2007). G * Power 3: A flexible statistical power analysis program for the social, behavioral, and biomedical sciences. Behav. Res. Methods.

[31] Wong S., Kenssous N., Hillier C., Pollmer S., Jackson P., Lewis S., Saif M. (2018). Detecting malnutrition risk and obesity after spinal cord injury: A quality improvement project and systematic review. Eur. J. Clin. Nutr..

[32] Lee J.H., Song C.H., Yum K.S., Kim K.S., Nam S.W., Han J.Y., Jeong G.W., Sun H.S. (2003). Age Associated Changes in Body Mass Index and Body Fat Distribution. J. Korean Acad. Fam. Med..

[33] Castro M.J., Apple D.F., Hillegass E.A., Dudley G.A. (1999). Influence of complete spinal cord injury on skeletal muscle cross-sectional area within the first 6 months of injury. Eur. J. Appl. Physiol. Occup. Physiol..

[34] Saxton S.N., Toms L.K., Aldous R.G., Withers S.B., Ohanian J., Heagerty A.M. (2021). Restoring perivascular adipose tissue function in obesity using exercise. Cardiovasc. Drugs Ther..

[35] Shojaei M.H., Alavinia S.M., Craven B.C. (2017). Management of obesity after spinal cord injury: A systematic review. J. Spinal Cord Med..

[36] Dolbow D.R., Credeur D.P., Lemacks J.L., Stokic D.S., Pattanaik S., Corbin G.N., Courtner A.S. (2020). Electrically induced cycling and nutritional counseling for counteracting obesity after spinal cord injury: A pilot study. J. Spinal Cord Med..

[37] Spungen A.M., Adkins R.H., Stewart C.A., Wang J., Pierson R.N., Waters R.L., Bauman W.A. (2003). Factors influencing body composition in persons with spinal cord injury: A cross-sectional study. J. Appl. Physiol..

[38] Smidowicz A., Regula J. (2015). Effect of nutritional status and dietary patterns on human serum C-reactive protein and interleukin-6 concentrations. Adv. Nutr..

[39] Prüss H., Tedeschi A., Thiriot A., Lynch L., Loughhead S.M., Stutte S., Mazo I.B., Kopp M.A., Brommer B., Blex C. (2017). Spinal cord injury-induced immunodeficiency is mediated by a sympathetic-neuroendocrine adrenal reflex. Nat. Neurosci..

[40] Evans D.C., Corkins M.R., Malone A., Miller S., Mogensen K.M., Guenter P., Jensen G.L., ASPEN Malnutrition Committe (2021). The Use of Visceral Proteins as Nutrition Markers: An ASPEN Position Paper. Nutr. Clin. Pract..

[41] Tanaka M., Momosaki R., Wakabayashi H., Kikura T., Maeda K. (2019). Relationship between nutritional status and improved ADL in individuals with cervical spinal cord injury in a convalescent rehabilitation ward. Spinal Cord.

